# The immunologic changes during different phases of intestinal anastomotic healing

**DOI:** 10.1002/jcla.23493

**Published:** 2020-07-21

**Authors:** Feng Zhang, Song Qiao, Chunqiao Li, Bo Wu, Stefan Reischl, Philipp‐Alexander Neumann

**Affiliations:** ^1^ Department of General Surgery Tongren Municipal People’s Hospital of Guizhou Medical University(GMU) Guizhou 554300 China; ^2^ Department of Surgery Klinikum rechts der Isar School of Medicine Technical University of Munich(TUM) Munich 81675 Germany

**Keywords:** anastomotic phases, cytokines, immune cells, intestinal surgery, would healing

## Abstract

Intestinal anatomosis is a complex and multicellular process that involving three overlapped phases: exudative phase, proliferative phase, and reparative phase. Undisturbed anastomotic healings are crucial for the recovery of patients after operations but unsuccessful healings are linked with a considerable mortality. This time, we concentrate on the immunologic changes during different phases of intestinal anastomotic healing and select several major immune cells and cytokines of each phase to get a better understanding of these immunologic changes in different phases, which will be significant for more precise therapy strategies in anastomoses.

## 1 INTRODUCTIONS

Intestinal anastomosis is a complicated and cellulous procedure and the barrier function of intestine is central to health and breaking down of the barrier is involved in wide varieties of clinical conditions.^1^ Successful anastomotic healings are crucial for the recovery of patients after surgeries but failed healings would result in fatal illnesses, prolonged hospitalizations, and even deaths.^2^ Despite an apparent appalling clinical need and comprehensive studies performed over past several years, basic and clinical researches targeted at knowing and improving anastomotic healing are still delaying because of multiple factorial challenges. Moreover, such complexity of this process presents challenges in development of proper animal models to study anastomotic healing and potential treatments, and further limiting translation from preclinical experiments to clinic.^3^ Therefore, finding an easy but feasible method to understand the whole procedure of anastomosis is of great significance.

As we know, intestinal anatomosis can be divided into three overlapped phases: exudative phase (other names called inflammatory phase or hemostasis phase), proliferative phase, and reparative phase (another name called remodeling phase).^4^, ^5^ To study the immunologic changes on each phase can help us to understand the differences among the three phases and get the whole picture of anastomosis as well. In this review, we focus on the contribution of several major immune cells and cytokines in each phase as follows (Table 1).

**TABLE 1:** Anastomotic phases and immune cells & cytokines

Phases	Immune Cells and Cytokines			
Exudative phase (Inflammatory/Hemostasis Phase): 1‐4 d	Platelets	Neutrophils	Platelet‐derived growth factor (PDFG)	Interleukin‐1 (IL‐1) family
Proliferative phase: 2‐14 d	Epithelial Cells	Macrophages	Vascular endothelial growth factor (VEGF)	Interferon gamma (IFN‐γ)
Reparative phase (Remodeling Phase): after 14 d	Fibroblasts	Lymphocytes	Basic fibroblast growth factor (bFGF)	Transforming growth factor beta (TGF‐β)

## 2 EXUDATIVE PHASE (INFLAMMATORY/HEMOSTASIS PHASE)

**FIGURE 1:**
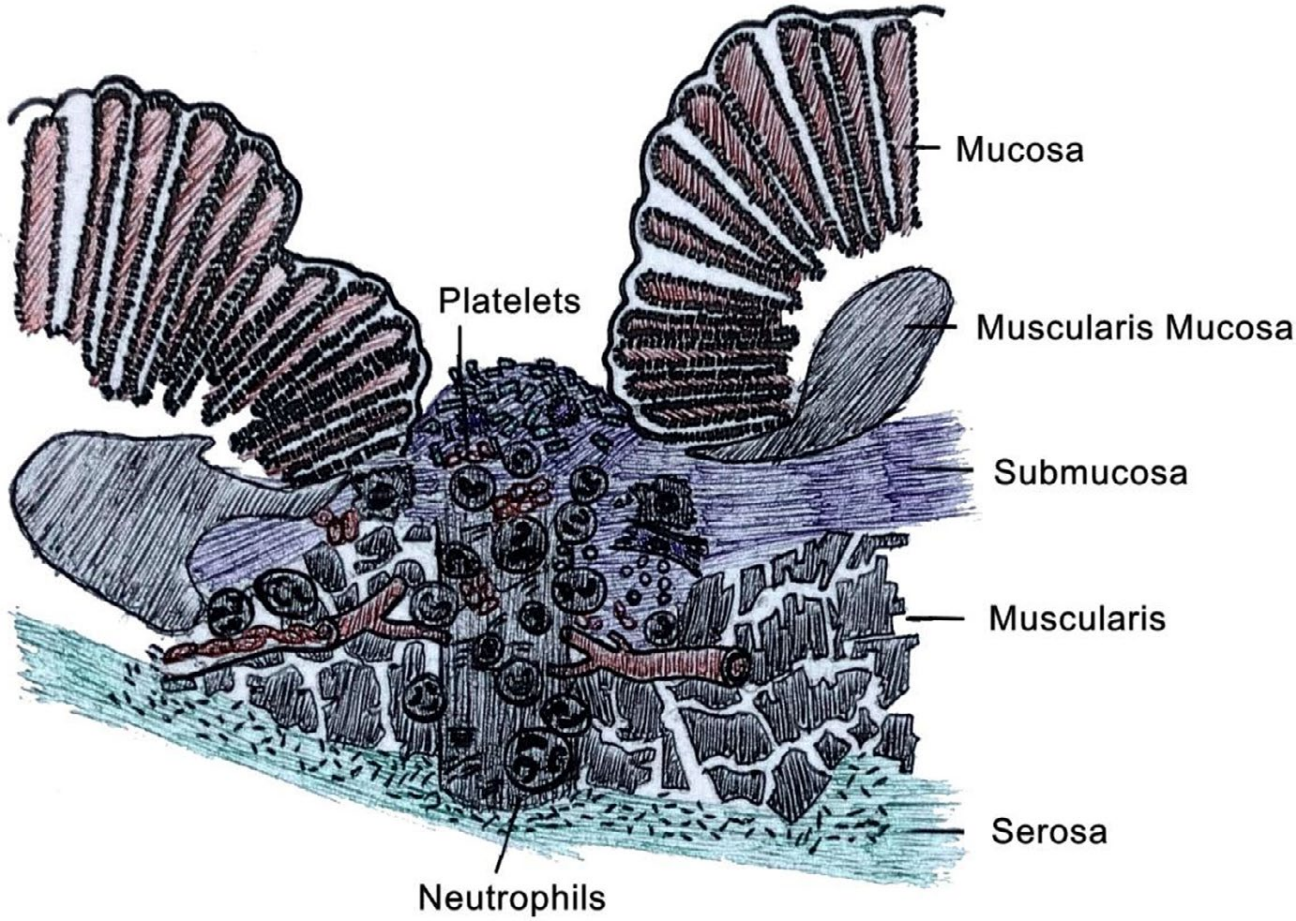
exudative phase

In the exudative phase, the immediate event when an injury occurs is platelet plugs that limits the bleeding and causes the release of a wide variety of immune cells and cytokines.^6^ This event begins the coagulation cascade and promotes expansion and recruitment of cells for the debridement of dead tissue that basically acts as a temporary wound closure mechanism.^7^ Within hours of the injury, neutrophils are drawn to and trapped in the platelet plugs in response to platelet‐derived growth factors (PDGF). They serve originally to phagocytize nonviable tissue and bacterial particles as well as use reactive oxygen species (ROS) to create a bacteria hostility environment.^8^ Neutrophils also provide an important pro‐inflammatory cytokine in interleukin‐1 (IL‐1), which is the first signal that warns surrounding cells to barrier damage and has double effects as a pro‐inflammatory cytokine and a stimulus for proliferation of keratinocytes. Keratinocytes release prestored IL‐1. PDGF together with pro‐inflammatory cytokines such as IL‐1 is significant in attracting neutrophils to the wound site to remove contaminating bacteria (Figure 1).^9^

### 2.1 Platelets

Platelets, small‐sized, complex non‐nucleated blood components, and first described over 100‐year ago, were conventionally conceived to purely play an important role in regulating hemostasis. However, there is increasing experimental and clinical evidence recognizes that platelets also have a crucial role in inflammation and immunization.^10^, ^11^

Although it is not completely understood yet, the immune function of platelets is a delicate balance between its regulation of hemostatic functions and its innate and adaptive immune responses.^12^

#### 2.1.1 The function of platelets

Platelets play an important role in the vessel and exist in circulation for 5‐7 days, primarily act as regulators of hemostasis.^13^ When vascular damage or injury, platelets become activated in the blood. Then adhere to the exposed extracellular matrix (ECM) and eventually form platelet plugs and consolidate clots.^14^ However, in addition to regulating hemostasis which is the well‐known function of platelets, some other potential functions of platelets have been declared, including the role in innate and adaptive immunity.^15^

The platelets’ interaction with immune cells such as neutrophils is central to start the immune response, and this response is functioned through the Toll‐like receptors (TLRs).^16^ The TLR family is made up of 13 recognized members, and 10 of them are expressed in humans. TLRs are key pattern recognition receptors of the innate immune system and are located either at the cell surface such as TLR‐1, TLR‐2, TLR‐4, TLR‐5, and TLR‐6, or in the intracellular such as TLR‐3, TLR‐7, TLR‐8, and TLR‐9.^17^ TLRs are expressed by cells comprising the dermis and epidermis, such as keratinocytes. The activation and timing of specific TLRs and the presence of conditions affecting TLR expression and activation determine whether TLR activation promotes or inhibits the wound healing process, leading to chronic wounds.^6^ Podoplanin is an endogenous ligand for C‐type lectin‐like receptor 2 (CLEC‐2), which is expressed on platelets, podoplanin/ CLEC‐2 signaling regulates keratinocyte migration via modulating E‐cadherin expression through RhoA signaling.^18^ Therefore, altering the regulation of keratinocyte migration by Podoplanin expressed on platelets will be a novel method to wound healing.

### 2.2 Neutrophils

Neutrophils are the most abundant immune cells to a new wound, and they are very active during the healing of wounds.^19^ It is known that neutrophils remove the debris in the early phase of anastomotic healing. Concurrent with the process of hemostasis, neutrophils represent the first cells to migrate to the wound bed, brought in by pro‐inflammatory signals such as IL‐1.^20^ Although neutrophils have a very short half‐life in blood both in mice and humans, pro‐inflammatory cytokines such as IL‐1 increase their lifespan, which may contribute to relieve the inflammation.^21^

Mouse models of recent studies have shown that in non‐aged, non‐impaired models, neutrophils depletion does not negatively affect the wound healing, but in impaired models of wound healing, such as diabetes and inflamed gut, neutrophils are badly required.^22^, ^23^, ^24^

#### 2.2.1 The function of neutrophils

Although neutrophils are not considered as an essential cell type in non‐impaired, non‐aged wound healing, they do complete a variety of functions that support the process.^22^, ^23^, ^24^

First of all, neutrophils protect from wound infection by endocytosing pathogenic agents, and killing them via releasing reactive antimicrobial proteins.^22^ Then with the process of degranulation, antimicrobial proteins can be released into the surrounding environment to kill extracellular animate things.^25^

In addition to clear pathogenic agents, neutrophils also regulate inflammation and generate immune cells to induce wound healing. In the injured environment, neutrophils have the ability to increase the expression of cytokines and chemokines as well as additional neutrophils.^26^ Neutrophils also show increased expression of cytokines that promote angiogenesis, such as vascular endothelial growth factor (VEGF), proliferation of fibroblasts, keratinocytes, such as IL‐1, and tissue remodeling, which are essential to the wound healing.^27^, ^28^, ^29^

### 2.3 Platelet‐derived growth factor （PDGF）

Platelet‐derived growth factor (PDGF) is a significant factor driving wound healing of actually almost all organs.^30^ Platelet‐derived growth factor is a significant mediator in the early phase of wound healing. It was discovered nearly 40 years, and it was found to have an important role in wound healing for almost 30 years as well, but studies into its physiological roles, functions, and structures are ongoing.^31^, ^32^, ^33^

There are five known members in the PDGF family: PDGF‐AA, PDGF‐BB, PDGF‐AB, PDGF‐CC, and PDGF‐DD.^34^ And PDGF‐BB is believed by researchers to be the most important and related member for wound healing.^35^, ^36^

#### 2.3.1 The function of platelet‐derived growth factor（PDGF）

Platelet‐derived growth factors stimulate the production of ROS during the exudative phase of wound healing.^37^ We understand that PDGF‐BB is the most effective factor that drives the first phase of wound healing.^35^, ^36^ Firstly, PDGF‐BB cure methods doubled the rate of complete re‐epithelialization of wound healing. Secondly, increased vessel formation was also found and was a significant part of the increased granulation tissue present in PDGF‐BB cured wound healing. It is more important that the remarkable increase in granulation tissue formation mediated by PDGF‐BB was fully reversible.^38^, ^39^

PDGF is also known for stimulating the chemotaxis of neutrophils and macrophages which are crucial in the inflammatory phase of wound healing and produce ROS via nicotinamide adenine dinucleotide phosphate oxidase (NADPH oxidase).^40^ PDGF could increase ROS production by stimulating the migration of immune cells and cytokines to the wound site and stimulate macrophages to produce growth factors which are critical for wound healing.^41^

### 2.4 Interleukin‐1 （il‐1） family

The innate immune system is the front line defense of our bodies. It is nondirected and unspecific, through the complement activation or the activation of innate immune receptors, the innate immune response is started.^42^, ^43^

Cytokines of the IL‐1 family play key roles in innate immunity. There are eleven members in this family: IL‐1α, IL‐1β, IL‐1RA, IL‐18, IL‐36Ra, IL‐36α, IL‐37, IL‐36β, IL‐36γ, IL‐38, and IL‐33. Some researchers also term them from IL‐1F1 to IL‐1F1 depending on the order of their discoveries.^44^

Though IL‐1 family is component the innate immune system, it is also influence on T‐cell functions. Thus, IL‐1 family is an important bridge between an early innate immune response and an adaptive immune response followed. Some studies also show that the IL‐1 family members (such as IL‐1β, IL‐33) impact the inflammation phase of wound healing as well.^45^, ^46^, ^47^

#### 2.4.1 The function of interleukin‐1 （IL‐1） family

IL‐1 family members are key inflammatory cytokines that generally act synergistically to amplify the inflammatory response. During wound healing, IL‐1 is expressed majorly by neutrophils and macrophages.^48^

Decreased inflammatory cell numbers and reduced epidermal thickness and fibrosis are found in the IL‐1R KO mice and wild‐type mice treated with IL‐1ra.^49^ Decreased inflammatory cytokines, myofibroblasts, and proliferating cells are found by injecting of IL‐1ra.^50^ Other members of the IL‐1 family (such as IL‐18 and IL‐33) are also involved to regulate the inflammatory and reparative response. IL‐18 participates in pro‐inflammatory signaling, while IL‐33 exerts cytoprotective effects.^51^ And the early use of the IL‐1Ra will inhibit the efficacy of IL‐1 in the inflammatory cascade and can prevent early granulation formation.^52^ These researches indicate that IL‐1 family is a significant indirect mediator of wound healing and that inhibition of IL‐1 signal could contribute to wound healing.

## 3 PROLIFERATIVE PHASE

**FIGURE 2:**
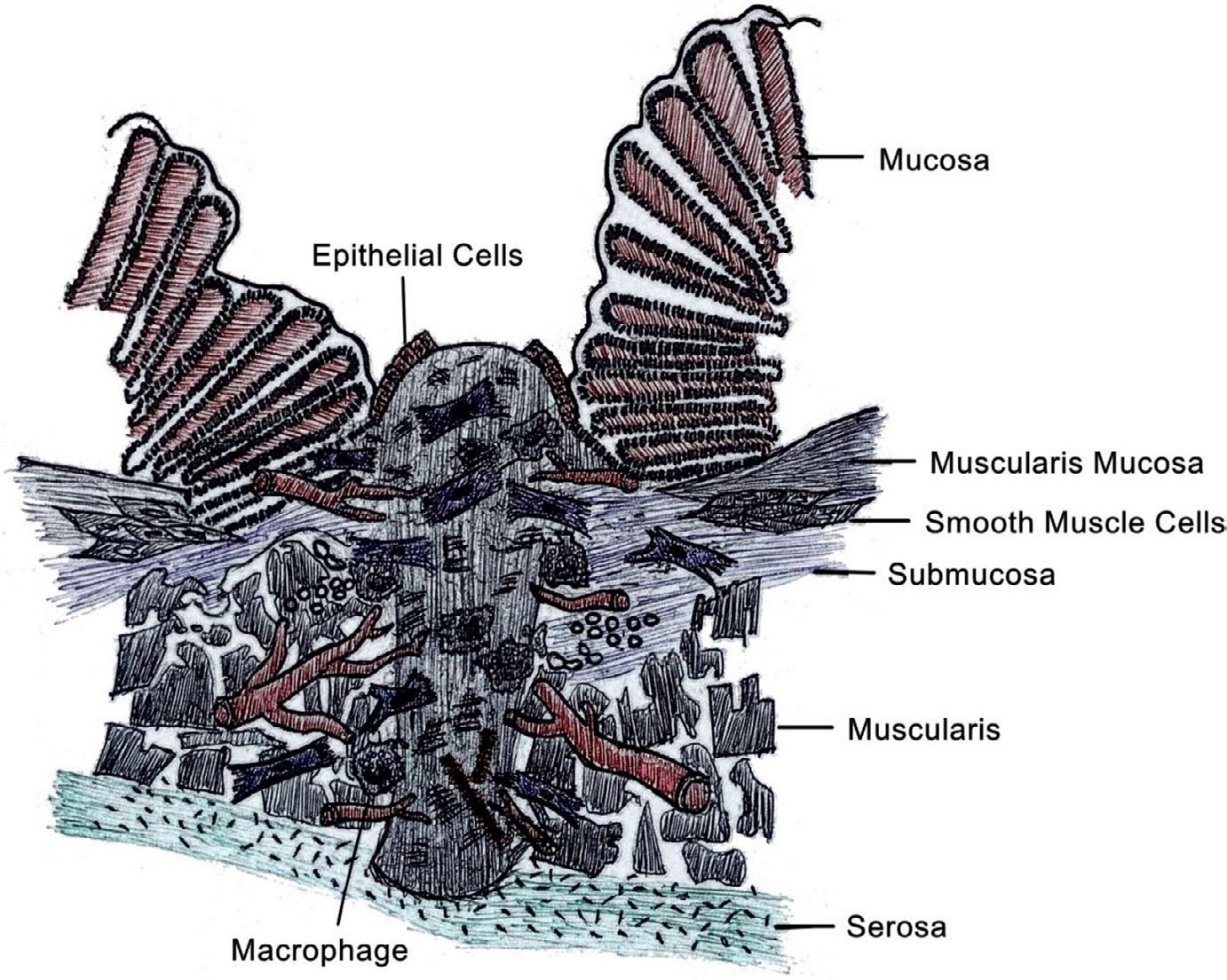
proliferative phase

In proliferative phase, the main focus of the healing process lies in covering the wound surface, angiogenesis, and epithelialization. Though there are overlaps between the wound healing phases, the capability to transit into the next phase will decide whether a wound heals suitably.^53^, ^54^ Epithelialization occurs early in wound repair shortly after injuring. And endothelial cells produce vascular endothelial growth factor (VEGF), which is crucial to restoring impaired angiogenesis process. Macrophages have numerous functions like hosting defense, promoting, and solving inflammation, removing apoptotic cells and supporting cell proliferation and tissue restoration after injury. Macrophages also play an indispensable role in a successful healing process through the synthesis of numerous potent growth factors such as VEGF, which promote cell proliferation. And under the control of regulating cytokines such as IFN‐γ, the synthesis of collagen, fibronectin, and other basic substances needed for wound healing serves for the closure of tissue gaps and the restoration and enhances the wound healing effects (Figure 2).^55^, ^56^

### 3.1 Epithelial cells

Intestinal epithelial cells, which line the inner face of the intestinal tract, have various significant functions,^57^ such as absorbing food substances, immune functions like cytokine secretion, via detoxification enzymes to work as barriers against xenobiotics and help the wound healing as well.^58^, ^59^

It is known that immediately after wound, coagulation and hemostasis are triggered in the injured issues. Humoral and cellular inflammatory phase follows with the formation of an immune barrier against invading microorganisms. And then, the wound healing mechanisms are later turned to tissue healing.^60^ Among the different processes in the proliferative phase, the epithelialization and angiogenesis are of particular significance. The epithelial cells have a special role, they make the growth and survival of new‐formed tissues possible, since all tissues are depending on blood supplies and this in turn depends on epithelial cells.^61^

#### 3.1.1 The function of epithelial cells

Epithelial cells allow organisms to keep internal homeostasis in changes of external environment and protect against infection. Some epithelial cells, take intestine epithelial cells for example, close breaches extremely fast and effectively limiting the entry of pathogens.^62^, ^63^

There are a quantity of epithelial signaling events come into play roles to mediate wound closure. Some recent studies have highlighted an important role of ROS signaling in coordinating wound healing.^64^ Though the oxygen has a key role in mediating wound repair is well known, the significance of cellular oxygen understanding in healing mechanisms is still a new area.^65^

We can also establish either animal models or epithelial cells in vitro culture to study their migratory behavior and their roles to play in wound healing, such as intestinal anastomotic healing and so on.^66^, ^67^, ^68^, ^69^

### 3.2 Macrophages

Similar to neutrophils, macrophages are also a significant part of the innate immune response to intestinal wound healing, partially because of their capability to start and solve inflammation and to contact with other innate and adaptive immune cells. As intestine has the largest pool of macrophages in the body,^70^ macrophages’ role in intestinal wound healing cannot be ignored.

Generally, macrophages can be categorized into three major subtypes: inflammatory monocytes which can be rapidly differentiate into activated macrophages (M1‐like phenotypes, mainly for pro‐inflammatory), tissue‐resident macrophages (M2‐like phenotypes, mainly for anti‐inflammatory), and regulatory macrophages.^71^ M2‐like phenotypes are believed more desirable for effective wound healing.^72^

#### 3.2.1 The function of macrophages

In all injuries, macrophages are significant players.^73^ They create an inflammation environment for clearing possible pathogens, resolve the inflammation when the pathogens are cleared, and are also participants in starting tissue remodeling.^74^ But macrophages contribute most to the proliferative phase of wound healing, especially M2‐like phenotypes’ role in mediating resolution of inflammation. M2‐like phenotypes are alternative activated cells that regulated by T helper type 2 (Th2).^75^

M2‐like phenotypes have the ability to self‐renew and can be long‐lived in body. Following the injury, M2 macrophages express adhesion molecules that recruit and guide multiple cell types. Moreover, M2 macrophages can replicate to double or triple their numbers, which are orchestrating in this wound healing process.^76^

Recent studies also show that macrophages take into effect to promote wound healing through WNT pathways which are firstly found as necessary pathways for tissue and organ development.^77^, ^78^ WNT ligands secreted by macrophages enhance the intestinal regeneration.^79^ Transfer of M2 macrophages will quicken the wound healing in TNBS‐treated (2, 4, 6‐trinitrobenzenesulfonic acid treated) mice via the activation of the WNT signaling pathway.^80^

### 3.3 Vascular endothelial growth factor （VEGF）

Vascular endothelial growth factor (VEGF) is an effective and selective mitogen for vascular endothelial cells and plays a significant role in Angiogenesis.^81^ It helps wound repair by adding the vascular permeability of local blood vessels, contributing the flow of inflammatory cells to the wound sites and adding the proliferation of endothelial cells which plays important roles in wound healing.^82^, ^83^

There are five members in VEGF family including VEFG‐A, VEFG‐B, VEFG‐C, VEFG‐D, and placenta growth factor (PLGF).^84^ Among them, VEGF‐A participates in the process of wound healing, its transcription and secretion will be elevated and reach the peak at the approximately 7th day after injury. And it mainly influences the proliferative phase.^85^

#### 3.3.1 The function of vascular endothelial growth factor （VEGF）

It is already known that a special property of VEGF is to add vascular permeability. VEGF was named vascular permeability factor for a time before its amino acid sequence was clear.^82^, ^83^ With further researches in VEGF, we know that VEGF is also a powerful and positive mediator for endothelial cells to format new blood vessels, such as proliferation.^86^ The blood vascular components depend on angiogenesis, in which new blood vessels appear approximately from 3rd to 7th day after injury. And then, capillary growth into the injured sites subsequently provides passages for nutrients and other mediators of the wound healing.^87^

While generally beneficial, VEGF also acts as a chemical attractant for invading pathogens, VEGF is a crucial cytokine for angiogesis, and it is also influenced by the bacterial infections, such as *Pseudomonas aeruginosa*, which may harmful to the wound healing.^88^ Some studies show a new concept called VEGF‐driven keratinocyte response,^89^, ^90^ although we still need to find more evidence for VEGF interaction with keratinocytes, it may give another perspective to wound healing therapies in clinic.^91^

### 3.4 Interferon gamma (IFN‐γ）

IFN‐γ is a main component in immune cell signaling and is a significant mediate protein for general immune responses. Its effects on cells are remarkable and have been found to regulate the expression of thousands human genes. Although IFN‐γhas functions of anti viral, it is more notable for stimulating and regulating the immune cells.^92^, ^93^

The innate and adaptive immune responses depend on controlled IFN‐γ expression. And present researches show that IFN‐γhas a significant role in the proliferation phase of wound healing via the mediation of the immune responses at the injury sites.^94^ Thus, it is of great significance to understand the pathways that mediate the expression of IFN‐γ.

#### 3.4.1 The function of interferon gamma (IFN‐γ）

IFN‐γ is basically secreted by CD4 + and NK cells, and it contributes mainly to the activation of immune cells and has relationships with both neutrophil recruitment and cell clearance.^95^, ^96^ As for its role in wound healing, some studies show that it can enhance the healing procedure and effects, such as IFN‐γ enhances the blood vascular regeneration and wound healing through significantly upregulated BST2 expression in both LEPCs and ECs and increased tube formation in LEPCs.^92^

As IFN‐γ is well known regarding its inhibitory effects on collagen synthesis by fibroblasts, Its role in wound healing remains controversial,^97^ such as IFN‐KO mice exhibited accelerated healing compared with WT mice, showing that IFN‐γ makes a negative contribution to the wound healing procedure.^98^

Therefore, further investigations are necessary to illuminate the effects of IFN‐γ therapy on wound healing, and it is also of great importance to clarify its optional dose.^99^

## 4 REPARATIVE PHASE (REMODELING PHASE)

**FIGURE 3:**
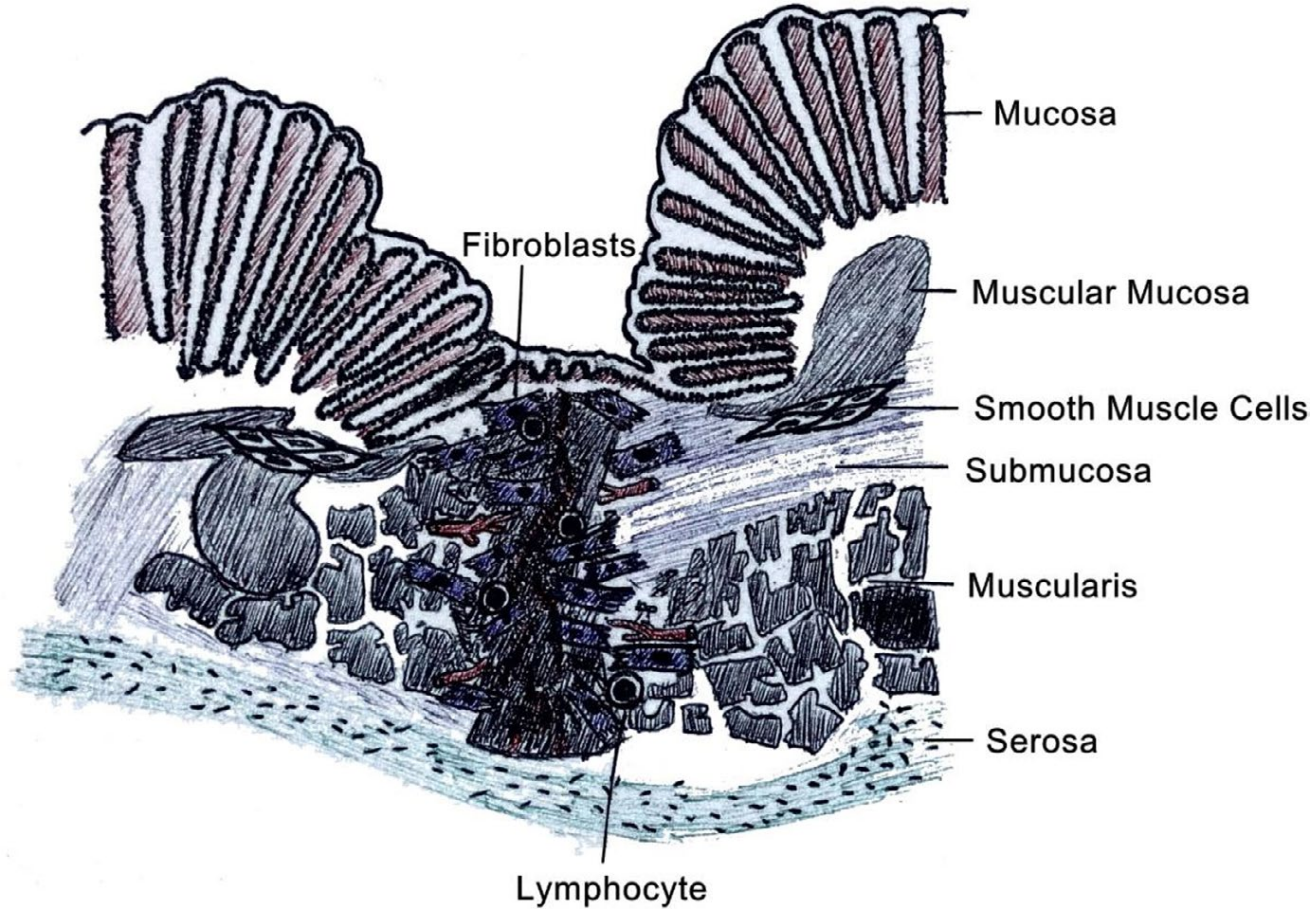
Reparative phase

The reparative phase is where the wound achieves maximum strength as it matures. It is characterized by wound contraction and collagen remodeling and the reorganization of the distinct layers of the intestinal wall.^54^ An essential feature of normal wound repair is the formation of granulation tissue, for instance, tissues containing fibroblasts, collagen and blood vessels, which means the hallmark of an established healing response. As we described above, macrophages are the key cells in proliferation phase, while fibroblasts are becoming the principle cells in remodeling phase.^100^ Net collagen synthesis will continue after wounding. The added rate of collagen synthesis during wound healing is from the increase of fibroblasts.^101^ The quality and appearance of the wound healing or repairing is mostly decided by this phase. Therefore, we should have a better management on this phase in order to get a better scar or scarless healing. In this part, we focus on four major cells and cytokines which may helpful in reparative phase (Figure 3).

### 4.1 Fibroblasts

Fibroblasts, mesenchymal cells, are present in many tissues in the body and play a major role in structural support.^102^ Since they have the ability to secrete and respond to cytokines, they also take part in the wound healing processes, especially in reparative phase.^103^

It has been more than 40 years since fibroblasts were first reported, much interest has concentrated on the control of them since that time. They have capability of changing during the wound healing processes to a contractile phenotype involved in adding ECM production and contraction in the process of wound healing.^104^, ^105^

#### 4.1.1 The function of fibroblasts

The activity of fibroblasts and their following differentiation is relied on the links of the action of growth factors, ECM components, and mechanical stress. Local proliferation and migration from adjacent tissues, especially near the vascular region, have generally been accepted as the mechanisms by which the tissue fibroblast numbers may increase.^106^, ^107^, ^108^

Rinkevich and his colleagues demonstrated the discovery of a “scarring fibroblast” that responsible for depositing the very majority of scar tissue in mice. They showed that these same cells could be reliably identified through expression of the marker CD26 and that ablation of these cells would reduce the scarring, though this also might delay the wound healing.^109^ In order to get a better understand of this “scarring fibroblast”, Plikus and his colleagues’ research illustrated that during the wound repairs, fat cells can be generated from activated fibroblasts which involved in wound contraction.^110^ Some other researches also demonstrated the lineage among fibroblasts involved in wound healing.^111^, ^112^

However, further studies are needed to fully clarify the contributions of different fibroblast lineages to wound healing, characterize the most specific subtype both in animals and human beings.

### 4.2 Lymphocytes

Lymphocytes are critical components of the adaptive immune responses, originally from the bone marrow, and can be mainly divided to three directions: mature into B lymphocytes; travel to thymus and develop into T lymphocytes; and stay primitive as Natural killer cells (NKCs).^113^ Among them, B lymphocytes develop into plasma cells which secrete antibodies. T lymphocytes can be further divided into CD4 + helper cells and CD8 + cytotoxic cells on the basis of their surface marker proteins. CD4 + cells can activate B lymphocytes in order to make B lymphocytes work properly. CD8 + cells have the ability to clear viral‐infected or dysfunctional cells.^114^

Adaptive immunity activation requires highly specific cooperation between antigen‐presenting cells and distinct antigen‐specific receptors on lymphocytes. Lymphocytes, especially T lymphocytes, play a significant regulatory role in wound healing through both the modulation function of fibrosis and its adaptive immune responses pathway.^115^, ^116^

#### 4.2.1 The function of lymphocytes

Lymphocytes have a regulatory role in normal wound healing through the secretion of lymphokines that are soluble protein factors produced by antigen‐stimulated lymphocytes and act as chemical messengers.^114^ Some studies have shown that lymphokines influence fibroblast activities and collagen synthesis which belong to the reparative phase or remodeling phase of wound healing.^117^

The process of lymphocytes activation that is stimulated by antigen‐presenting cells mainly happens in the lymph nodes and spleen. Then, activated‐lymphocytes are transported to the periphery through the lymphatic vessels and arterial vessels. Some researchers believed that lymphocytes are crucial to competent wound healing since they perform significant regulatory functions during wound healing as well as their important roles in adaptive immune responses.^118^, ^119^

In order to examine lymphocytes relationship with adaptive immunity, researchers have found that T lymphocytes promote the wound healing via endogenous vascular endothelial growth factor receptor 1 tyrosine kinase (VEGFR1‐TK) pathway.^120^ And they also secrete the lymphokines to regulate the healing of the epithelium and protect barrier function of intestinal epithelial cells.^121^ Another research group even use neutrophil‐lymphocyte ratio and platelet‐lymphocyte ratio to predict the effect of wound healing in reconstruction.^122^

### 4.3 Basic fibroblast growth factor (bFGF）

Fibroblast growth factors are a very big family consisted of many homologous peptides, such as Acidic fibroblast growth factor (aFGF or FGF‐1), Basic fibroblast growth factor (bFGF or FGF‐2) and Keratinocyte growth factor (KGF or FGF‐7).^123^, ^124^ Among FGF family, bFGF’s ability to accelerate the process of both acute and chronic wound healing has been already proved and it is mainly produced by fibroblasts, macrophages, and endothelial cells.^125^

The bFGF is a multiple potential glycoprotein that promotes various cells such as fibroblasts, keratinocytes, and endothelial cells. Because of its mitogenic and angiogenic characteristics, the bFGF plays an important role in inducing tissue remodeling and wound healing.^126^, ^127^

#### 4.3.1 The function of basic fibroblast growth factor（bFGF）

The bFGF is a powerful mitogen and chemical attractant for endothelial cells and fibroblasts and stimulates the metabolism and growth of the ECM, which are very important for wound healing. In some animal experiment, bFGF‐knockout mice showed delayed healing of skin injury.^128^, ^129^

And bFGF is widely accepted and used in accelerating wound healing in clinical treatment. Some studies show bFGF is not only helpful for wound repair, but also improve the scar quality and regeneration.^130^, ^131^ The practice and action of bFGF in scar management are highly significant both in understanding scarless wound healing in the laboratory and fulfilling minimally invasive concept in the clinic.^132^ Accelerating wound healing improves the quality of healing and alleviates the scar.^133^ Those researches imply a feasible anti‐scarring effect of bFGF during wound healing.

Although human recombinant bFGF is used for wound healing far and wide nowadays, the problem of its short half‐life still remains to be sorted.^134^, ^135^, ^136^

### 4.4 Transforming growth factor beta (TGF‐β)

Transforming growth factor‐β (TGF‐β) is a multiple functional cytokine that plays a key role in wound healing and in tissue repairing. TGF‐β is found in almost all tissues in body, and it is mainly produced by infiltrating cells, like platelets, macrophages, and lymphocytes.^137^, ^138^ Thus, after the injury, these cells are becoming potential sources of TGF‐β.

Generally, the production and activation of TGF‐β will stimulate the production of various ECM proteins and will decelerate the breakdown of those proteins as well. TGF‐β contributes to wound healing through these actions. Under ideal circumstances, we hope the wound heals to the restoration of normal tissue architecture or at least to a scar less tissue.^139^, ^140^ Therefore, exploration of TGF‐β activity in scarless wound healing, and understanding of TGF‐β function in scarless wound healing is a very promising field which is also of great significance in improving healing qualities in clinical scenarios, such as in inflamed colons and diabetic wounds.

#### 4.4.1 The function of transforming growth factor beta（TGF‐β）

Several growth factors are involved in wound repair, while central to wound healing is TGF‐β which is of particular significance for almost all phases of this process, especially in the remodeling phase.^141^ TGF‐β exerts multi‐effects on wound healing by regulating cell proliferation, differentiation, ECM production, and mediating the immune response as well. TGF‐β is a cytokine which is secreted by several different cell types involved in wound healing and has different effects.^142^

Many different cell types are involved in wound healing, such as epithelial cells, fibroblasts, and macrophages are shown to be responsive to TGF‐β.^142^ TGF‐β promotes monocyte chemotaxis and growth factor production. Moreover, it promotes the regenerative maturation of keratinocytes and recruits fibroblasts into the wound bed. And its most significant function is stimulating the collagen production by fibroblasts, though the procedure of collagen production is also involves other cytokines like IFN‐γ.^142^, ^143^

Several studies offered the evidences that TGF‐β was present in the healing wound, and suggested that TGF‐β might be an important marker of the wound healing procedures. We also noted that there were an increased fibroblasts and an obvious increase in collagen deposition with the application of TGF‐β. These studies might imply that TGF‐β is a potential pharmacological agent to accelerate the wound healing.^144^, ^145^, ^146^, ^147^

## 5 CLOSING REMARKS AND OUTLOOK

Successful intestinal anastomotic healing managements require a thoroughly understanding of wound healing processes and the related factors like immune cells and cytokines that play important roles on them. This review tries to illustrate the immunologic changes in different healing phases and cover 12 significant immune cells and cytokines, average 4 immune cells, and cytokines in each phase. As it is mentioned at the beginning, the three anastomotic healing phases are an overlapping process, thus an immune cell or cytokine may influence multiple or even the whole healing procedure. But we assign it in one phase according to the greatest impact where it has.

Immune cells and cytokines are critical for coordinating multiple cell types in intestinal anastomotic healing that makes the wound repairing possible. Effective wound healing should be guided by strict regulation of these regulators as well as a good repair circumstance that supports their actions.

Nowadays, although many promising biomarkers are used in different sample collecting ways, we should pay attention to the consistent implementation and efficacious follow‐up therapeutics as well.

Another impressive finding is that some cytokines such as basic fibroblast growth factor（bFGF） and transforming growth factor beta （TGF‐β） which have the anti‐scar effect should be seen and paid attention. It may never be possible to eliminate the risk of an injury, whether artificial (like anastomotic operations) or accidental (like inflammatory or traumatic diseases), but we should try our best to repair it with minimally invasion and continue to expand our medical armamentarium that helps us to get a better and more successful healing.
